# Amino acids suppress macropinocytosis and promote release of CSF1 receptor in macrophages

**DOI:** 10.1242/jcs.259284

**Published:** 2022-02-21

**Authors:** Zachary I. Mendel, Mack B. Reynolds, Basel H. Abuaita, Mary X. O'Riordan, Joel A. Swanson

**Affiliations:** 1 Department of Microbiology and Immunology, University of Michigan Medical School, Ann Arbor, MI 48109, USA; 2 Graduate Program in Immunology, University of Michigan Medical School, Ann Arbor, MI 48109, USA

**Keywords:** Macropinocytosis, CSF1R, Amino acids, Macrophages, CSF1, Macropinosomes, Mouse

## Abstract

The internalization of solutes by macropinocytosis provides an essential route for nutrient uptake in many cells. Macrophages increase macropinocytosis in response to growth factors and other stimuli. To test the hypothesis that nutrient environments modulate solute uptake by macropinocytosis, this study analyzed the effects of extracellular amino acids on the accumulation of fluorescent fluid-phase probes in murine macrophages. Nine amino acids, added individually or together, were capable of suppressing macropinocytosis in murine bone marrow-derived macrophages stimulated with the growth factors colony stimulating factor 1 (CSF1) or interleukin 34, both ligands of the CSF1 receptor (CSF1R). The suppressive amino acids did not inhibit macropinocytosis in response to lipopolysaccharide, the chemokine CXCL12, or the tumor promoter phorbol myristate acetate. Suppressive amino acids promoted release of CSF1R from cells and resulted in the formation of smaller macropinosomes in response to CSF1. This suppression of growth factor-stimulated macropinocytosis indicates that different nutrient environments modulate CSF1R levels and bulk ingestion by macropinocytosis, with likely consequences for macrophage growth and function.

## Abstract (precis)

**Summary:** Nine amino acids suppress CSF1-stimulated macropinocytosis by promoting the loss of CSF1 receptor, indicating a regulation by nutrients of growth factor receptor signaling.

## INTRODUCTION

Macropinocytosis is an actin-driven cellular process in which cells internalize relatively large volumes of fluid into plasma membrane-derived vesicles known as macropinosomes. It has been implicated in antigen presentation, cell growth and metabolic regulation (Sallusto et al., 1995; Commisso et al., 2013; Palm et al., 2015; Yoshida et al., 2015). Macropinocytosis may occur constitutively or following stimulation by growth factors, chemokines or microbial products (Stow et al., 2020). In macrophages, the growth factor colony stimulating factor 1 (CSF1) stimulates macropinocytosis (Racoosin and Swanson, 1989). Although extensive work has defined the molecular mechanisms involved in the formation, internalization and trafficking of macropinosomes (Stow et al., 2020), the regulation of macropinocytosis has been relatively understudied.

Macropinocytosis provides a mechanism for nutrient uptake that can support growth for tumor cells and lymphocytes (Commisso et al., 2013; Palm et al., 2015; Charpentier et al., 2020). Following stimulation of macrophages with CSF1, macropinocytosis delivers extracellular leucine into lysosomes to activate the mechanistic target of rapamycin complex 1 (mTORC1), a nutrient sensing complex that functions as a central regulator of cell growth (Yoshida et al., 2015; Condon and Sabatini, 2019). As macropinocytosis provides nutrients to the cell, we hypothesized that nutrients themselves may regulate this process.

Several studies have indicated such regulation. Glucose depletion increases macropinocytosis in some cancer cell lines, but not in non-transformed cells (Kim et al., 2018). Besterman et al. (1983) showed that constitutive pinocytosis in rabbit alveolar macrophages decreased in the presence of essential amino acids; however, the mechanism of pinocytosis or its inhibition by amino acids was not determined. Specific single amino acids can alter a variety of cellular processes (Chantranupong et al., 2015), including macropinocytosis (Lee et al., 2019). In *Dictyostelium*, arginine, lysine and glutamate can individually upregulate macropinocytosis (Williams and Kay, 2018). In pancreatic ductal adenocarcinoma tumors, glutamine depletion enhances macropinocytosis by potentiating epidermal growth factor receptor signaling (Lee et al., 2019). Here, we examined the effects of single amino acids on macropinocytosis in murine bone marrow-derived macrophages (BMM) and discovered that some amino acids suppress CSF1-stimulated macropinocytosis by promoting the loss of CSF1 receptor (CSF1R).

## RESULTS

### Nine amino acids suppress macropinocytosis

**Fig. 1.**
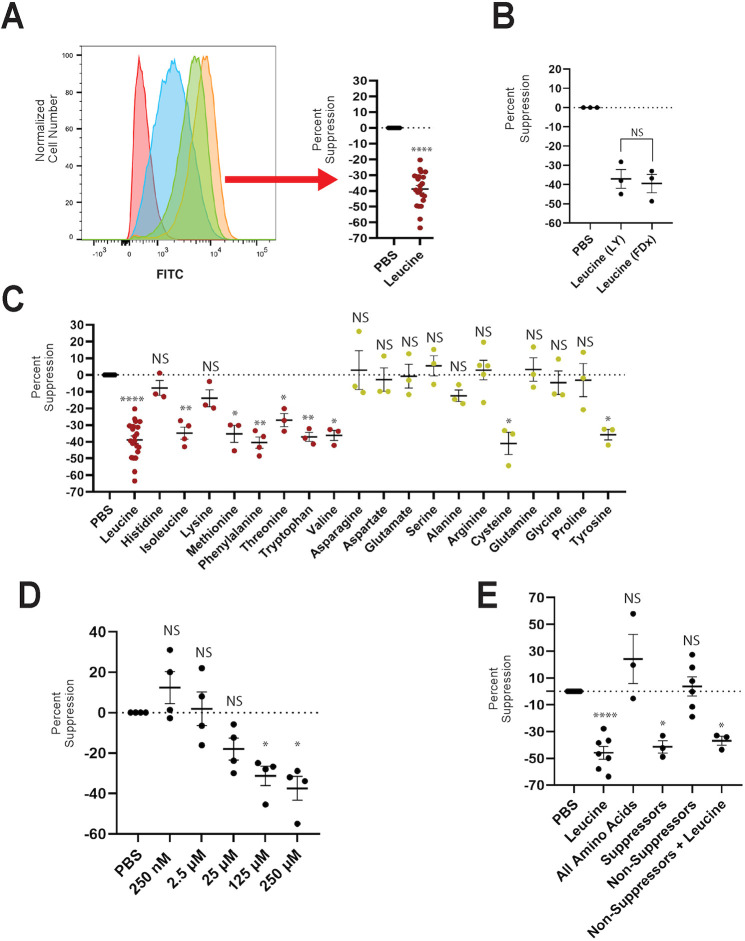
**Amino acids suppress macropinocytosis.** (A) BMM were incubated for 30 min in PBS with or without leucine, then for 60 min with FDx and CSF1. Solute accumulation was analyzed by flow cytometry. Representative results (left) showing cells incubated with FDx at 4°C (red), FDx at 37°C (blue), FDx+CSF1+leucine at 37°C (green) and FDx+CSF1 at 37°C (orange). The flow data (right) is displayed as ‘percent suppression’, in which FDx accumulation by cells incubated with CSF1 and leucine is compared with that in CSF1 alone (reference value). Each data point is calculated using the median of the fluorescence distributions for a single experiment. (B) BMM were incubated for 30 min in either PBS containing 0.25 mM leucine or PBS alone. Cells were then incubated for 30 min with CSF1 plus either FDx or LY. Percent suppression comparing cells incubated in leucine to those in PBS alone. (C) Cells were incubated for 30 min in PBS with or without the indicated amino acid, then for 60 min with FDx, CSF1 and the indicated amino acid. Percent suppression comparing macropinocytosis in CSF1 in PBS alone with that in CSF1 plus the indicated amino acid. Red, essential amino acids; yellow, non-essential amino acids. (D) Percent suppression of macropinocytosis of cells incubated in PBS with or without leucine ranging in concentration from 250 nM to 250 µM. Cells were incubated for 30 min in PBS with or without the indicated concentration of leucine, then for 60 min with FDx, CSF1 and leucine. (E) Cells were incubated for 30 min in the indicated mixture of amino acids, then for 60 min with FDx and CSF1. Percent suppression by mixtures of amino acids compared with PBS alone. The non-suppressor group included histidine, lysine, asparagine, aspartate, glutamate, serine, alanine, arginine, glutamine, glycine and proline. The suppressor group included leucine, isoleucine, methionine, phenylalanine, threonine, tryptophan, valine, cysteine and tyrosine. The ‘all amino acid’ group included all 20 amino acids. pH was adjusted to 7.2-7.4 for all conditions. *N*≥3 independent experiments. Bars indicate mean±s.e.m. Statistics were performed using two-tailed ratio paired *t*-tests for all experiments comparing the experimental condition with the PBS control, using the raw values as opposed to the relative values, which are shown. *P<0.05, **P<0.01, ****P<0.0001. NS, not significant.

We first examined the effect of leucine on macropinocytosis, as leucine has a well-characterized role as an activator of mTORC1 (Hara et al., 1998; Wolfson and Sabatini, 2017). BMM were deprived of CSF1 overnight, then incubated for 30 min in phosphate-buffered saline (PBS) with or without 0.25 mM leucine, a physiologically relevant concentration (Takach et al., 2014). Cells were then incubated for 60 min with 70 kDa fluorescein-isothiocyanate dextran (FDx), a specific marker for macropinocytosis (Berthiaume et al., 1995; Li et al., 2015), with or without CSF1 or leucine, before analysis by flow cytometry. Leucine inhibited CSF1-stimulated uptake of FDx by 40% (Fig. 1A). To further explore the physiological relevance of this finding, we performed these experiments in the presence of albumin, the major protein in circulation (Palm, 2018), as well as glucose. Although both bovine serum albumin (BSA) and glucose slightly increased macropinocytosis, leucine still suppressed macropinocytosis in those conditions (Fig. S1).

In dendritic cells, FDx endocytosis is mediated in part by the mannose receptor (Sallusto et al., 1995). In BMM, FDx is a valid probe for fluid-phase endocytosis (Berthiaume et al., 1995). However, as macrophages sometimes express high levels of the mannose receptor (Fiani et al., 1998), we sought to confirm that leucine was specifically affecting fluid-phase macropinocytosis and not mannose receptor-mediated endocytosis. Lucifer Yellow (LY) is a fluid-phase probe, the internalization of which is not mediated by the mannose receptor (Swanson et al., 1985; Berthiaume et al., 1995; Sallusto et al., 1995). CSF1-stimulated uptake of LY was measured in the presence or absence of leucine. Similar to FDx, CSF1-stimulated uptake of LY was reduced in the presence of leucine (Fig. 1B). This indicates that mannose-receptor-mediated endocytosis did not contribute to the accumulation of FDx by macrophages.

We next asked whether other amino acids could suppress macropinocytosis. We performed similar experiments as in Fig. 1A, incubating BMM in PBS containing each of the other 19 amino acids that comprise proteins. To be consistent with the leucine experiments, the concentration for each amino acid was 0.25 mM. We defined any amino acid that reduced total macropinocytic uptake by at least 20% as a suppressor, and one that did not as a non-suppressor. Nine of the twenty amino acids were suppressors (Fig. 1C). Most of the essential amino acids were suppressors, with the exception of histidine and lysine. The non-essential amino acids were non-suppressors, with the exception of cysteine and tyrosine. We next sought to determine the minimal concentration by which leucine suppresses by macropinocytosis. To this end, cells were incubated in PBS with or without leucine at different concentrations and CSF1-stimulated uptake was quantified. Maximal suppression of macropinocytosis occurred at leucine concentrations greater than 125 µM, with intermediate suppression at concentrations ∼25 µM. No suppression occurred at concentrations below 2.5 µM (Fig. 1D).

Combined suppressors and non-suppressors behaved like the individual amino acids: macropinocytosis was suppressed in cells incubated with the nine suppressive amino acids but was not suppressed when incubated with the 11 non-suppressive amino acids (Fig. 1E). However, when macrophages were incubated in a mixture containing all twenty amino acids, no suppression was observed (Fig. 1E). From this we hypothesized that either the non-suppressive amino acids were dominant in the ‘all amino acid’ mixture, or particular non-suppressive amino acids could inhibit the action of the suppressive amino acids. To address this, cells were incubated in a mixture containing leucine plus the 11 non-suppressive amino acids. Leucine was sufficient to suppress macropinocytosis (Fig. 1E). This refutes the hypothesis that one or more non-suppressive amino acids can inhibit suppression. We cannot yet explain why leucine or the other suppressors were not dominant when all amino acids were present.

### Amino acids selectively suppress CSF1R-dependent macropinocytosis

**Fig. 2.**
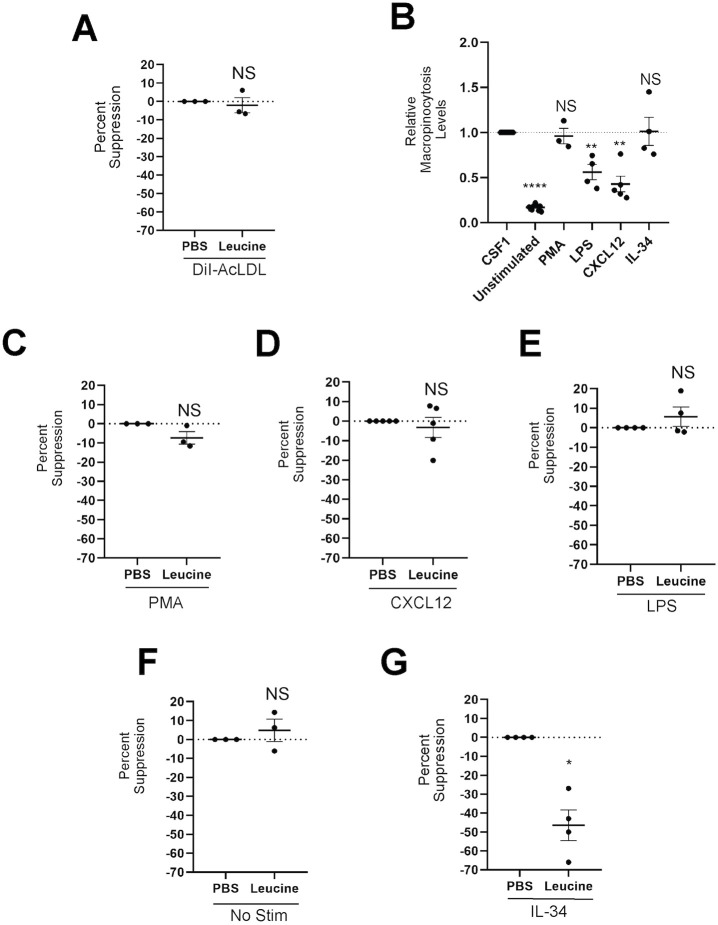
**CSF1R is necessary for suppression of macropinocytosis by leucine.** (A) BMM were incubated for 30 min in PBS with or without leucine, then for 15 min with DiI-AcLDL or CSF1 plus FDx before analysis by flow cytometry. Shown is the percent suppression comparing cells incubated in leucine with those in PBS alone. (B-G) BMM were incubated for 30 min in PBS with or without leucine, then for 60 min with FDx and CSF1, CXCL12, PMA, LPS, IL34 or no stimulation. Solute accumulation was analyzed by flow cytometry. (B) Relative levels of macropinocytosis are determined by normalizing the median fluorescence values in each condition to that in response to CSF1. (C-G) Shown is the percent suppression by leucine, comparing levels of macropinocytosis in cells incubated with PMA (C), CXCL12 (D), LPS (E), without stimulant (F) or with IL34 (G) with that of cells incubated in the indicated stimulant without leucine. *N*≥3 independent experiments. Each data point represents an independent experiment. Bars indicate mean±s.e.m. Statistics were performed using two-tailed ratio paired *t*-tests for all experiments comparing the experimental condition with the PBS control, using the raw values as opposed to the relative values, which are shown. *P<0.05, **P<0.01, ****P<0.0001. NS, not significant.

To examine whether this amino acid-dependent suppression of uptake is present in other forms of endocytosis, we used leucine as a model suppressive amino acid and evaluated its effect on receptor-mediated endocytosis. We measured uptake of fluorescent acetylated low density lipoprotein (AcLDL), which binds to class A scavenger receptors and is internalized in clathrin-coated pits (Whitman et al., 2000). Cells were allowed to internalize either DiI-labeled AcLDL or FDx plus CSF1 in the presence or absence of leucine. Leucine did not inhibit uptake of DiI-AcLDL (Fig. 2A), suggesting that leucine specifically downregulates macropinocytosis. Macropinocytosis can be induced in macrophages by stimuli other than CSF1, including lipopolysaccharide (LPS) (Zanoni et al., 2011), the chemokine CXCL12 (Lou et al., 2014) and the tumor promoter phorbol 12-myristate 13-acetate (PMA) (Swanson, 1989). To determine whether macropinocytosis is suppressed generally by amino acids, we compared the effects of leucine on macropinocytosis induced by CSF1, PMA, LPS or CXCL12. All increased macropinocytosis, with CXCL12 and LPS stimulating less than PMA and CSF1 (Fig. 2B). Leucine failed to suppress constitutive pinocytosis in unstimulated BMM, as well as macropinocytosis in response to PMA, CXCL12 or LPS (Fig. 2C-F). To test whether amino acid-dependent suppression was specific to CSF1R signaling, we used interleukin 34 (IL34), which signals through CSF1R (Lin et al., 2008) to promote macrophage differentiation, growth and survival (Chihara et al., 2010; Boulakirba et al., 2018). IL34 stimulated macropinocytosis to the same extent as CSF1 (Fig. 2B). IL34-stimulated macropinocytosis was inhibited by leucine (Fig. 2G), suggesting that amino acid-dependent suppression of macropinocytosis is specific to CSF1R signaling.

### Suppressive amino acids promote loss of CSF1R

**Fig. 3.**
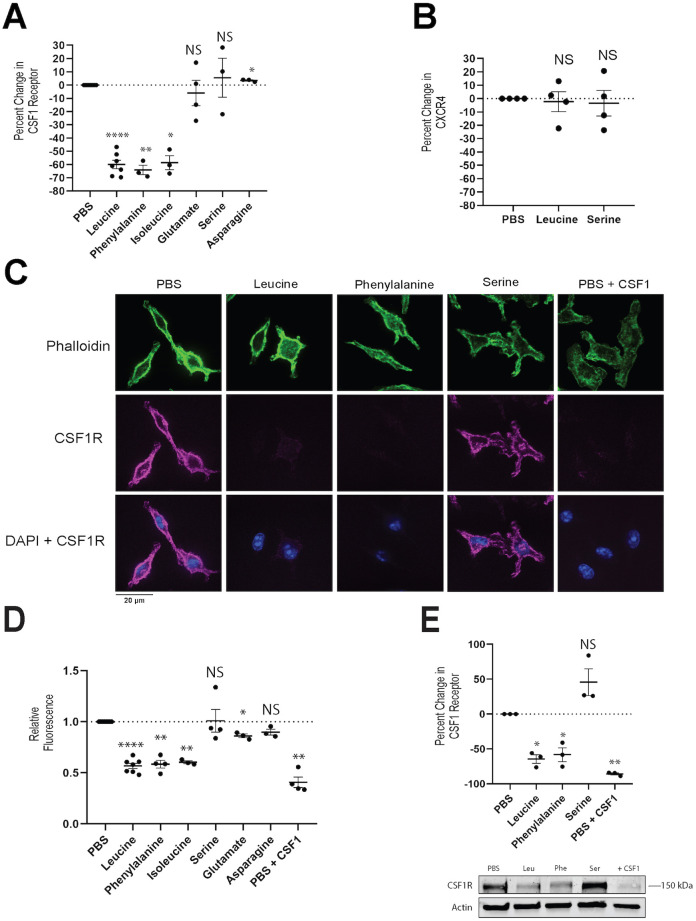
**Suppressive amino acids reduce CSF1R levels.** (A,B) BMM were incubated for 60 min in either PBS containing the indicated amino acid or in PBS alone. Cells were collected, stained using anti-CSF1R antibody or anti-CXCR4 antibody, and then analyzed by flow cytometry to measure cell surface receptor levels. Percent reduction in either CSF1R or CXCR4 was calculated by comparing the mean fluorescence of the population incubated with leucine with that incubated in PBS alone. (A) The percent change in CSF1R levels compared with PBS for three suppressive amino acids (leucine, phenylalanine, and isoleucine) and three non-suppressive amino acids (glutamate, serine, and asparagine). (B) The percent change in CXCR4 compared with PBS for cells incubated in either leucine or serine. (C,D) BMM were incubated for 60 min in either PBS alone or PBS containing the indicated amino acid. Cells were permeabilized and stained using anti-CSF1R antibody to visualize total CSF1R. Actin was labeled using Phalloidin-iFluor 488, and nuclei were labeled using DAPI. Representative confocal images (C) and quantification of the microscopy data (D) showing the average CSF1R fluorescence of cells incubated in the various amino acid conditions. Data were normalized to the PBS condition. (E) BMM were incubated in either PBS containing the indicated amino acid or PBS alone for 60 min. Cells were lysed and blotted for CSF1R. Quantification of the blots from three independent experiments (top). CSF1R levels are normalized to the actin loading control. Representative western blot gel (bottom). *N*≥3 independent experiments. Each data point represents an independent experiment. Bars indicate mean±s.e.m. Statistics were performed using two-tailed ratio paired *t*-tests for all experiments comparing the experimental condition with the PBS control, using the raw values as opposed to the relative values, which are shown. *P<0.05, **P<0.01, ****P<0.0001. NS, not significant.

To begin to define a mechanism by which leucine suppresses IL34- and CSF1-stimulated macropinocytosis, we measured the effects of amino acids on cell surface expression of CSF1R. Compared with the PBS control, the suppressors leucine, phenylalanine and isoleucine significantly reduced the cell surface levels of CSF1R. The non-suppressors serine, asparagine and glutamate did not (Fig. 3A). As a control for specificity, we measured cell surface levels of CXCR4, the receptor for CXCL12 (Bleul et al., 1996). As CXCL12-stimulated macropinocytosis was not sensitive to leucine, we expected that incubation with leucine would not reduce levels of CXCR4. Cell surface CXCR4 was unaffected by the presence of leucine (Fig. 3B), indicating that leucine and other suppressive amino acids specifically reduce surface levels of CSF1R.

To determine whether CSF1R was sequestered or degraded in response to suppressive amino acids, BMM were incubated in PBS with or without a suppressor (phenylalanine, leucine or isoleucine), or a non-suppressor (serine, glutamate or asparagine). Cells were then fixed, permeabilized and stained to detect both intracellular and surface CSF1R. As a control for CSF1R degradation, cells were incubated in PBS+CSF1, as CSF1 promotes the internalization and degradation of CSF1R (Lee et al., 1999). Cells were imaged by confocal microscopy and processed using a CellProfiler™ analytical pipeline, which allowed quantification of levels of CSF1R on a per cell basis for each of the conditions examined. Cells incubated with CSF1 exhibited ∼60% reduction in CSF1R levels (Fig. 3C,D). Cells incubated with a suppressor exhibited significant reductions in CSF1R levels, whereas those incubated with a non-suppressor did not (Fig. 3C,D). The microscopy data were confirmed using western blotting. (Fig. 3E). Thus, our data suggest that CSF1R levels are reduced in response to suppressive amino acids.

**Fig. 4.**
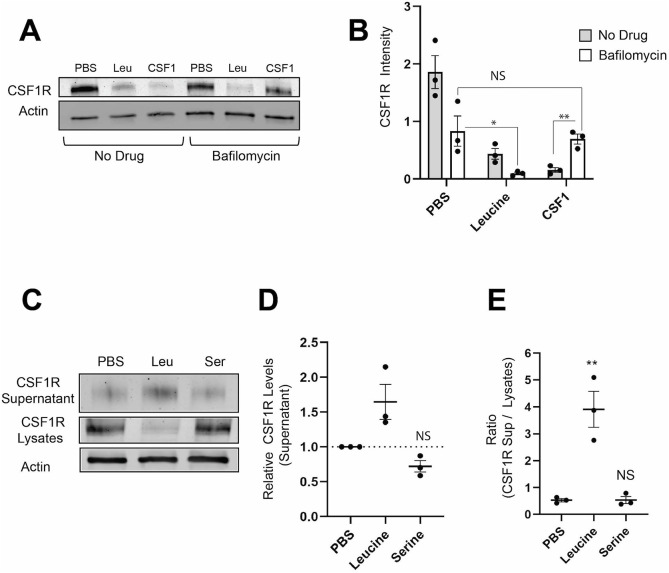
**Leucine promotes secretion of CSF1R.** (A,B) Cells were either pre-treated for 60 min with 500 nM Baf or left untreated, then incubated for 60 min in either PBS containing the indicated amino acid or in PBS alone ±Baf. Cells were lysed and probed for CSF1R. Representative western blot (A) and quantification of western blots from three independent experiments (B) of cells showing CSF1R levels in cells with or without Baf. CSF1R levels are normalized to actin loading controls. (C-E) BMM were incubated 60 min in either PBS containing the indicated amino acid or PBS alone. Supernatants were collected and concentrated using Amicon Ultra-2 30 K filters. Cells were lysed. Both concentrated supernatants and cell lysates were probed for CSF1R. (C) Representative western blot. (D) Quantification of the supernatant data from three independent experiments showing the relative amount of CSF1R in the supernatants of cells normalized to the PBS condition. For leucine *P*=0.08. (E) Ratio of CSF1R levels in the supernatant compared with the levels in the cell lysate. CSF1R levels in each condition are normalized for amount loaded. Each data point represents an independent experiment. Bars indicate mean±s.e.m. Statistics were performed using two-tailed ratio paired *t*-tests for all experiments. On figures showing relative amounts, statistics are performed using the raw values as opposed to the relative values. *P<0.05, **P<0.01. NS, not significant.

To test the hypothesis that CSF1R was being degraded, we incubated cells with leucine in the presence of bafilomycin A1 (Baf). Baf is macrolide antibiotic which functions as a potent inhibitor of the vacuolar ATPase, preventing lysosomal acidification and acid hydrolase-dependent protein degradation (Bowman et al., 1988). If leucine promotes lysosomal degradation of CSF1R, we would expect an increase in CSF1R levels in the Baf+leucine condition compared with the leucine alone condition. As a positive control for the effect of Baf on lysosomal degradation of CSF1R, cells were incubated with CSF1, which is known to promote lysosomal degradation of CSF1R, with or without Baf. We observed a significant increase in CSF1R levels, comparing the CSF1+Baf-treated cells to the CSF1 alone cells (Fig. 4A,B). Contrary to our hypothesis, however, Baf did not increase CSF1R in the leucine-treated cells, which suggests that leucine does not promote lysosomal degradation of CSF1R (Fig. 4A,B).

As leucine appeared to promote neither sequestration nor degradation of CSF1R, we hypothesized that leucine was promoting the release of CSF1R from the macrophages. To test this, we assayed for the presence of CSF1R in the supernatants of cells incubated in either PBS alone, PBS with the suppressor leucine or PBS with the non-suppressor serine. Significantly higher levels of CSF1R were detected in the supernatant of cells incubated in leucine compared with those incubated in serine or PBS alone (Fig. 4C-E). Thus, our data suggest that leucine promotes the release of CSF1R from macrophages.

**Fig. 5.**
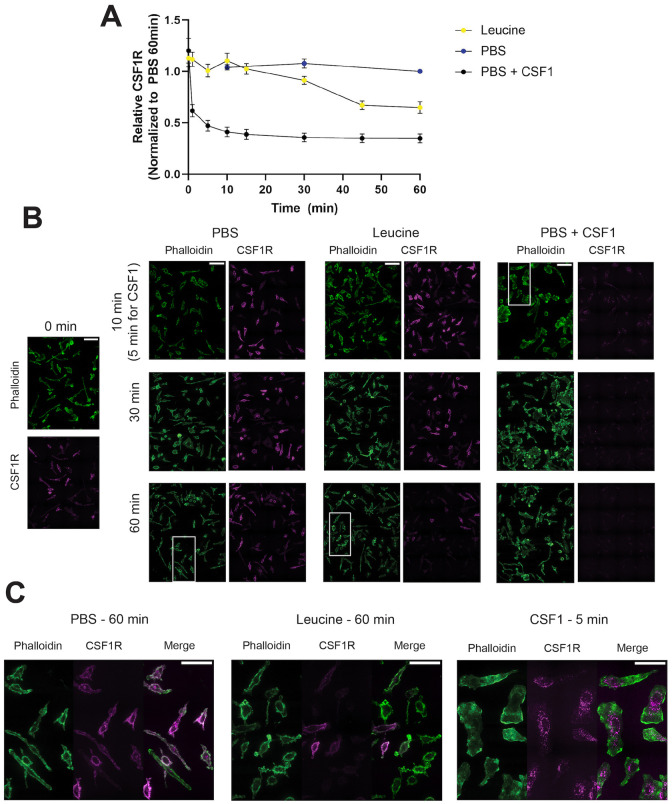
**Leucine-induced loss of CSF1R is a slow process.** (A-C) Time course was performed in which BMM were incubated for 1 min to 60 min in either PBS alone, PBS containing leucine, or PBS+CSF1. Cells were permeabilized and stained using anti-CSF1R receptor antibody. Actin was labeled using Phalloidin-iFluor 488, and nuclei were labeled using DAPI. Cells were imaged by confocal microscopy. (A) Quantification of the time course. All data were normalized to the 60 min PBS condition. Bars indicate mean±s.e.m. (B) Representative images showing population level changes of cells incubated in the different conditions. White boxed areas represent the area shown in greater detail in C. The actin and CSF1R signals were set to the same intensities in the different conditions. Scale bars: 50 μm. (C) Enlarged micrographs of BMM in the different conditions. The CSF1R signals were set to the same intensities between the PBS±leucine conditions, but the CSF1R signal was greatly enhanced in the CSF1 condition to highlight the punctate intracellular localization of CSF1R. Actin signals were set to the same intensity between conditions. Scale bars: 30 μm. *N*=3 independent experiments.

To interrogate the dynamics of CSF1R release, we measured CSF1R levels of cells incubated in PBS with or without leucine at varying times from 1 min until 60 min. We also incubated cells in PBS with CSF1 to compare these dynamics with a degradative process. As expected, cells incubated in PBS alone exhibited constant levels of CSF1R throughout the time course, whereas incubation with CSF1 caused rapid internalization and degradation of CSF1R. In contrast, cells incubated in leucine maintained steady levels of CSF1R for the first 15 min of incubation, followed by a slight decrease from 15 min to 30 min, and finally a large decrease from 30 min to 45 min (Fig. 5). CSF1R levels stayed constant from 45 min to 60 min (Fig. 5). Moreover, as further evidence that leucine promotes release rather than internalization and degradation of CSF1R, we did not detect redistribution of CSF1R into punctate vesicles in the leucine condition, which was evident when CSF1R was downregulated by incubation in CSF1 (Fig. 5).

### Leucine reduces the size of macropinosomes

**Fig. 6.**
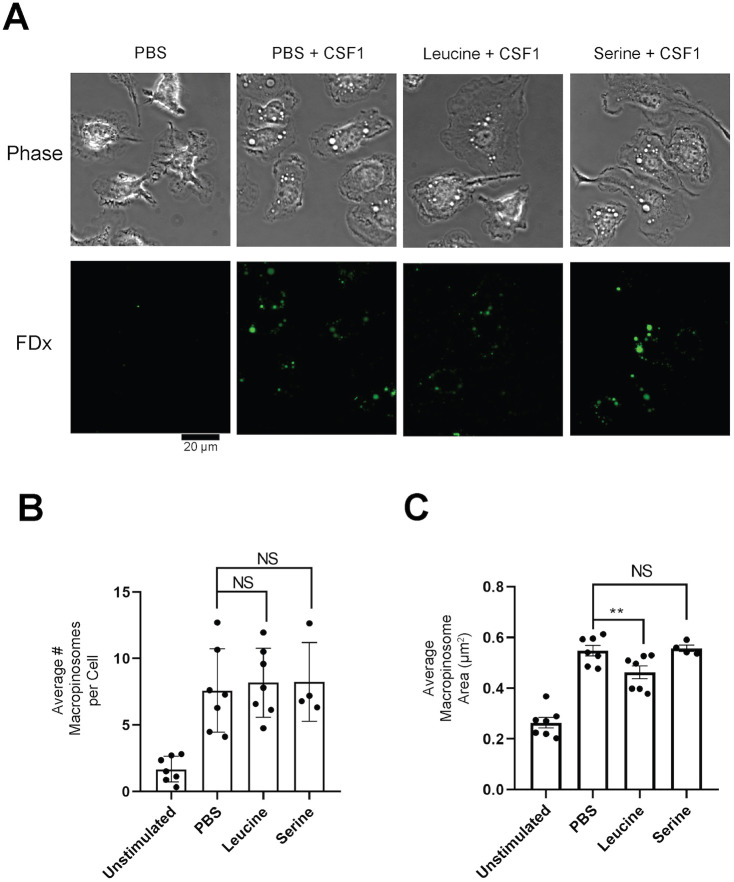
**BMM incubated with leucine generate smaller macropinosomes.** (A-C) BMM were incubated in PBS with or without the indicated amino acid for 30 min. FDx and CSF1 were added to the cells for 5 min. After washing the cells and incubating them in PBS containing Hoechst dye to label nuclei, cells were imaged for 4 min. (A) Representative images of macrophages either left unstimulated, with CSF1, with CSF1 and leucine, or with CSF1 and serine. (B) The average number of macropinosomes per cell is shown for the indicated conditions. (C) The average area of the macropinosomes in the indicated conditions. *N*≥4 independent experiments. Each data point represents a single experiment. Bars indicate mean±s.e.m. Statistics were performed using two-tailed ratio paired *t*-tests for all experiments comparing the experimental condition to the PBS control. **P<0.01. NS, not significant.

Lastly, to identify the mechanism of reduced solute accumulation by suppressive amino acids, we examined cells microscopically in conditions with or without suppressive amino acids. In live cell imaging of cells incubated in CSF1 or CSF1+leucine, we could not discern any obvious differences in ruffling or the process of macropinosome formation. This was likely due to the wide range of morphologies that characterize macropinocytosis (Quinn et al., 2021). Instead, we quantified the numbers and sizes of macropinosomes formed in response to CSF1. Cells were incubated for 30 min with or without leucine or serine, then were pulsed for 5 min with CSF1 and FDx. Because macropinosomes shrink and fuse shortly after closing into the cell, it was necessary to image them for morphometry after only brief pulses with FDx to best approximate their initial sizes. Earlier work from this lab (Racoosin and Swanson, 1993) and others (Maxson et al., 2021) showed that 1-5 min pulsed macropinosomes are enriched in markers of early endosomes, including Rab5. The cells were quickly washed and then imaged. An analytical pipeline was created using CellProfiler™ to quantify macropinosome numbers and sizes in the micrographs (Fig. 6A). The number of macropinosomes generated did not change significantly in any of the conditions (Fig. 6B), but cells incubated with leucine made significantly smaller macropinosomes compared with cells incubated with serine or PBS alone (Fig. 6C). This indicated that the decreased FDx accumulation observed in the flow cytometry data resulted from the formation of smaller macropinosomes. From this, we hypothesized that reducing CSF1R levels would result in smaller macropinosomes. To lower CSF1R levels another way, we incubated cells in CSF1 for 60 min, which lowers CSF1R levels in BMM significantly (Fig. 3). CSF1-treated cells and untreated control cells were pulsed for 5 min with CSF1 and FDx, then washed, fixed, imaged and analyzed in the CellProfiler™ pipeline. Cells incubated in CSF1 exhibited fewer and smaller macropinosomes compared with those incubated in PBS alone (Fig. S2). Thus, macrophages form smaller macropinosomes when CSF1R levels are decreased by exogenous molecules.

## DISCUSSION

The modulation of CSF1R expression described here suggests roles for amino acids in the regulation of macrophage physiology. CSF1R signaling is crucial for macrophage proliferation, survival and differentiation (Stanley and Chitu, 2014). Many exogenous molecules have been shown to modulate CSF1R (Guilbert and Stanley, 1984; Sester et al., 1999). CSF1 promotes the internalization and degradation of CSF1R, mainly via clathrin-coated vesicles (Lee et al., 1999; Lou et al., 2014), whereas LPS induces secretion of factors that downregulate CSF1R (Guilbert and Stanley, 1984). The present study shows that suppressive amino acids promote release of CSF1R, resulting in the formation of smaller macropinosomes and consequently less ingestion of extracellular solutes and nutrients.

The secretion of surface receptors in microvesicles has been shown in the context of an oncogenic form of the epidermal growth factor receptor, EGFRvIII, and of CCR5, the co-receptor for HIV-1 (Mack et al., 2000; Al-Nedawi et al., 2008). With regards to EGFRvIII secretion, microvesicles containing EGFRvIII can be internalized by cells lacking EGFRvIII, which transfers oncogenic activity to the cells lacking the mutant receptor (Al-Nedawi et al., 2008). Future studies aim to elucidate whether CSF1R is secreted in microvesicles or shed from the cell surface in another form.

*In vivo*, an imbalance of suppressive and non-suppressive amino acids in different nutrient environments, such as nutrient-replete healthy tissue or nutrient-deficient tumor microenvironments, could regulate surface expression of CSF1R, with consequent effects on macrophage growth, differentiation and function. Tumor-associated macrophages (TAMs) are anti-inflammatory and tumor promoting (Noy and Pollard, 2014). One of the molecules responsible for polarizing macrophages to this subset is CSF1 (Fleetwood et al., 2007). As the presence of TAMs is associated with poor prognosis in many cancer types (Zhang et al., 2012), therapies that modulate CSF1R signaling have garnered attention (Cannarile et al., 2017). The regulation described here may be relevant to macrophage differentiation in tumors.

The nine suppressors of CSF1R macropinocytosis were predominately essential amino acids, thus the detection system may be directed toward these amino acids. However, two non-essential amino acids, cysteine and tyrosine, suppressed macropinocytosis and two essential amino acids, histidine and lysine, did not. Cysteine and tyrosine require or substantially rely on an essential amino acid for their synthesis. Tyrosine requires phenylalanine, and cysteine substantially relies on methionine for its synthesis through the trans-sulfuration pathway (Choi and Colo, 2019; Sbodio et al., 2019). Thus, the requirement of essential amino acids for synthesis of cysteine and tyrosine may explain why they are included among the suppressors.

How suppressive amino acids promote release of CSF1R remains unknown, but the process may involve known metabolic sensors of amino acids. General control non-depressible-2 (GCN2; encoded by *EIF2AK4*) kinase senses uncharged tRNA molecules, which are abundant under amino acid starvation, and in turn phosphorylates eIF2α, resulting in a global downregulation of protein synthesis (Battu et al., 2017). Similarly, in amino acid-deficient environments, mTORC1 is dissociated from lysosomes, preventing its function as a metabolic hub to promote various anabolic processes (Efeyan et al., 2012). Inhibition of mTORC1 in human trophoblasts was recently shown to enhance macropinocytosis (Shao et al., 2021). GCN2 or mTORC1 may regulate CSF1R expression at the cell surface. Either the detection system or the mechanism of CSF1R release must be responsive to the composition of amino acids in the environment, as suppression is lost when all 20 amino acids are present.

The mechanisms regulating macropinosome size in metazoan cells are largely unknown. Unlike phagocytosis, in which the dimensions of a particle determine the size of the phagosome, there is no structure to guide macropinosome formation. Rather, macropinosomes are self-organized structures that assemble from cell surface ruffles that close into cups, which then close into macropinosomes (Swanson and Yoshida, 2019; Stow et al., 2020). What is known about the regulation of macropinosome size largely comes from genetic studies. In *Dictyostelium*, an Akt (PkbA), and an SGK (PkbR1), were shown to regulate macropinosome size, with PkbA/PkbR1 double knockouts forming smaller macropinosomes (Williams et al., 2019). Also in *Dictyostelium*, the RasGAP Neurofibromin was shown to regulate macropinosome size, with knockout mutants forming larger macropinosomes (Bloomfield et al., 2015). This study reveals exogenous regulation of macropinosome size by amino acids, resulting from a decrease in cell surface CSF1R levels.

If amino acids in the environment modulate growth factor receptor levels in other cell types, then this could have implications for the regulation of cell growth in tissues and the upregulation of macropinocytosis in some kinds of cancer cells. Accordingly, constitutive macropinocytosis in tumor cells may be due to loss of feedback inhibition by amino acids of growth factor-related signals. A more thorough understanding of the effects of nutrient environments on growth factor function could guide the design of therapies.

## MATERIALS AND METHODS

### Materials

RPMI-1640, fetal bovine serum (certified; FBS), GlutaMAX, penicillin-streptomycin (P/S), 70 kDa FDx, LY CH, DiI-AcLDL, unlabeled AcLDL, BSA, goat serum, DAPI solution (used at 1:10,000 dilution), paraformaldehyde, 0.25% Trypsin-EDTA with Phenol Red, Prolong™ Diamond Antifade Mountant, DPBS, Hoechst 33342, CellTracker™ Red CMTPX Dye, Halt™ Protease Inhibitor Cocktail and phycoerythrin CXCR4 antibody (2B11; diluted 1:1000) were purchased from Thermo Fisher Scientific. IL34 and recombinant mouse CSF1 were purchased from R&D Systems. HEPES, 2-mercaptoethanol, sucrose, Amicon Ultra-2 ml centrifugal filters and all amino acids were purchased from Sigma-Aldrich. Baf from *Streptomyces griseus* was purchased from Cell Signaling Technology. PMA, allophycocyanin anti-CSF1 receptor antibody (ab210247; diluted 1:1000) for immunofluorescence and flow cytometry, Phalloidin-iFluor 488 (diluted 1:10,000) and recombinant anti-CSF1 receptor antibody (ab221684; diluted 1:1000) for western blotting were purchased from Abcam. CXCL12 was purchased from Peprotech. LPS from *Salmonella* *typhimurium* was purchased from List Biological Laboratories. We purchased 35 mm dishes with 14 mm coverglass from MatTek Corporation. IRDye 680RD goat anti-rabbit IgG (#926-68071) and IRDye 800CW goat anti-mouse IgG (#926-32210) secondary antibodies for western blotting were purchased from LI-COR Biosciences and were diluted 1:10,000.

### Bone marrow macrophage isolation and culture

Macrophages were generated from C57BL6/J mice (Jackson Laboratory). Both male and female mice between the ages of 3 and 12 months were used. Bone marrow flushed from mouse femurs was differentiated into macrophages by culturing for 5 days in RPMI supplemented with 20% FBS, 50 ng/ml recombinant CSF1, 1% glutamax, 0.1% P/S and 37 µM 2-mercaptoethanol. Macrophages were detached using cold PBS lacking calcium and magnesium, and then 3-4×10^6^ cells/ml were frozen in the culture media described above with 10% DMSO and stored in liquid nitrogen. All animal-related procedures were approved by the University of Michigan Committee on Use and Care of Animals.

### Cell culture and stimulation

PBS used for all cell incubations contained the following ingredients: 0.90 mM calcium chloride, 0.49 mM magnesium chloride, 2.67 mM potassium chloride, 1.47 mM potassium phosphate monobasic, 137.93 mM sodium chloride, 8.06 mM sodium phosphate dibasic, containing 15 mM HEPES buffer (pH 7.2). For macropinocytosis assays, 1E6 BMM were plated on 60 mm treated dishes in RPMI containing 10% FBS, 1% glutamax, 0.1% P/S, 50 ng/ml CSF1 and 37 µM 2-mercaptoethanol. The medium was replaced with fresh medium 4 h after seeding. At the end of the following day, the medium was aspirated and replaced with RPMI containing 10% FBS, 1% glutamax and 0.1% P/S. The following day, cells were incubated with 300 ng/ml CSF1 and 0.5 mg/ml FDx or 0.5 mg/ml LY. For LPS treatments, cells were pretreated with 100 ng/ml LPS for 30 min, followed by addition of FDx. For PMA treatments, cells were pretreated for 15 min with 100 nM PMA, followed by addition of FDx. For IL34 and CXCL12 treatments, cells were treated with FDx and 100 ng/ml IL34 or 50 nM CXCL12.

For flow cytometry-based measurements of cell surface receptor levels, cells were plated on 60 mm untreated dishes in RPMI containing 10% FBS, 1% glutamax, 0.1% P/S, 37 µM 2-mercaptoethanol and 300 ng/ml CSF1. The medium was replaced with fresh medium 4 h after seeding. The experiments were performed the following day. Cells were stained with either 10 ng/µl anti-CSF1 receptor antibody or anti-CXCR4 antibody. For assays examining receptor-mediated endocytosis, cells were incubated for 15 min with 5 µg/ml DiI-AcLDL with or without 50 µg/ml unlabeled Ac-LDL, followed by rinsing and analysis by flow cytometry.

For western blot experiments, 2E6 BMM were seeded in 60 mm tissue culture-treated dishes in RPMI containing 10% FBS, 1% glutamax, 0.1% P/S, 37 µM 2-mercaptoethanol and 50 ng/ml CSF1. The medium was aspirated and replaced with fresh medium 4 h later. At the end of the following day the medium was aspirated and replaced with RPMI containing 10% FBS, 1% glutamax and 0.1% P/S overnight. For experiments involving Baf, the overnight medium was aspirated and replaced with fresh medium containing 500 nM Baf. For conditions not receiving Baf, the medium was replaced with fresh medium. Cells were pre-treated in Baf for 60 min followed by another 60 min in the assay conditions.

### Flow cytometry-based assays

For measuring macropinocytosis, BMM were seeded in 60 mm tissue culture dishes at 1E6/dish. On the day of the experiment, cells were washed 4× with PBS, then incubated in PBS containing 0.25 mM of the specified amino acid(s) or in buffer alone for 30 min at 37°C. Then 70 kDa FDx and either CSF1, LPS, CXCL12, IL34 or PMA were added for 60 min at 37°C. To remove cells from the dish, 0.25% trypsin-EDTA was added to the cells for 3 min at 37°C, at which point RPMI containing 10% FBS was added to the cells. Cells were removed from the dish by gentle scraping.

For measuring cell surface receptor levels, BMM were seeded in untreated 60 mm culture dishes at 2E6/dish. To begin the experiment, cells were washed 4× with PBS, and then incubated for 60 min in PBS containing 0.25 mM of the specified amino acid, or in PBS alone at 37°C. Cells were removed from the dishes by gentle scraping. Cells were pelleted by centrifugation at 500 ***g*** for 10 min and then resuspended in 100 µl of PBS. Then 1 µg of anti-CSF1 receptor antibody or anti-CXCR4 antibody was added to the cells.

For measuring receptor-mediated endocytosis, BMM were seeded in 60 mm tissue culture dishes at 1E6/dish. On the day of the experiment, cells were washed 4× with PBS, and then incubated for 30 min at 37°C in PBS only, or PBS containing 0.25 mM of the specified amino acid. Following this, cells were incubated with 5 µg/ml DiI-AcLDL, with or without 50 µg/ml unlabeled AcLDL, for 15 min at 37°C. Cells were washed with PBS. To remove cells from the dish, 0.25% trypsin-EDTA was added to the cells for 3 min, followed by the addition of RPMI containing FBS. Cells were removed from the dish by gentle scraping. All flow cytometric analysis was carried out using either a BD LTRFortessa or BD Canto (Becton-Dickenson).

### Microscopy

For quantifying macropinosome area and number in live cells, 6E4 BMM were seeded in a MatTek dish and cultured as detailed above. Cells were washed 4× with PBS and incubated in PBS containing 0.25 mM of the specified amino acid or PBS alone for 30 min at 37°C. CSF1 and FDx were added to the cells for 5 min, after which the cells were washed with PBS, placed in PBS containing Hoechst 33342 (1000×) and imaged for 4 min. Images were acquired on a Nikon TE300 inverted microscope equipped with a mercury arc lamp, Plan-Apochromat 60×, 1.4 NA objective, cooled digital CCD camera (Photometrics Coolsnap HQ^2^), temperature-controlled stage set at 37°C and a DAPI-FITC-Texas Red dichroic mirror (Chroma Technology). For each field of view, phase-contrast, 400 nm excitation 455 nm emission, and 490 nm excitation 535 nm emission images were taken using Metamorph Image Analysis Software (Molecular Devices).

For quantifying macropinosome area and number in fixed cells, 1E6 BMM were seeded on 18×18 mm glass coverslips placed in a 35 mm dish and cultured as detailed in the text. Following the 30 min incubations, the media was aspirated and replaced with PBS containing FDx, Hoechst 33342 and CSF1. Following a 5 min pulse, cells were fixed using fixation buffer 1 [20 mM HEPES (pH 7.4), 2% paraformaldehyde, 4.5% sucrose, 70 mM NaCl, 10 mM KCl, 10 mM MgCl_2_, 2 mM EGTA, 70 mM lysine-HCl] at room temperature for 15 min. Cells were washed for 15 min then incubated for 30 min at room temperature with CellTracker™ Red CMTPX Dye (1000×). Cells were then washed, mounted and imaged 48 h later using a Nikon X1 Yokogawa Spinning Disk Confocal microscope equipped with an iXon Ultra 888 camera, with Plan Apo 100×/1.45 oil objective.

For quantifying CSF1 receptor levels, 1E6 BMM were seeded on 18×18 mm glass coverslips placed in a six-well plate. The cells were cultured in the same manner as for standard macropinocytosis assays. Cells were incubated for 60 min in their respective conditions and then fixed for 15 min using 4% paraformaldehyde in PBS. The cells were then washed for 15 min using PBS (containing 0.1% Triton X), then incubated for 45 min in blocking buffer (PBS containing 0.1% Triton X, 5% BSA w/v, 10% goat serum v/v). CSF1R antibody, DAPI and Fluorescein-Phalloidin dyes were then added for 30 min at room temperature. Cells were washed and mounted in Prolong Diamond, then imaged at least 48 h later using a Nikon X1 Yokogawa Spinning Disk Confocal microscope equipped with an iXon Ultra 888 camera, with Plan Apo 100×/1.45 oil objective.

### Western blotting

For performing western blots on cell lysates, medium was aspirated and 100 µl of lysis buffer (1% NP-40 lysis buffer with 1× complete mini protease inhibitor) was added to the cells. Cells were scraped, collected and incubated on ice for 15 min. After centrifugation, 4× Laemmli buffer with β-mercaptoethanol was added to the supernatant. Cell lysates were separated by SDS-PAGE, and protein was transferred to a nitrocellulose membrane by a semidry transfer method. The membrane was blocked with blocking buffer (5% BSA w/v and 0.1% Tween-20 v/v in PBS) for 30 min at room temperature. Primary antibodies diluted in blocking buffer were added at 4°C overnight. Membranes were washed with PBS and then incubated with secondary antibodies (LI-COR #926-68071 and #926-32210; diluted 1:10,000) in blocking buffer for 30 min, followed by a wash in PBS. Western blots were visualized using the LI-COR Odyssey infrared imaging system. Gels were quantified according to the ImageJ densitometric gel analysis protocol for 1D gels (https://imagej.nih.gov/ij/docs/menus/analyze.html#gels).

For performing western blots on cell supernatants, protease inhibitor (100×) was first added to the collected supernatants, which were then spun at 1000 ***g*** for 10 min at 4°C. Supernatants were concentrated using Amicon Ultra 30K centrifugal filter devices. In brief, supernatants were spun at 4000 ***g*** for 15 min using a Sorvall ST 16R centrifuge with a swinging bucket rotor. The flow-through was discarded and the eluate was obtained by centrifugation at 1000 ***g*** for 2 min. Then 4× Laemmli buffer with β-mercaptoethanol was added to the concentrated supernatants and the western blotting was performed as described above.

### Quantifying macropinocytosis and CSF1R using ilastik and CellProfiler

To measure the frequency and size of macropinosomes on a single-cell basis using microscopy, we developed an automated image analysis pipeline which uses the open-source software ilastik™ and CellProfiler™. Phase contrast images were processed to generate cell masks using the ilastik Pixel Classification workflow. This assigns the probability that pixels in an image fit user-defined criteria in a Random Forest machine learning model. In these experiments, 10% of images were used for training, sampling 3-4 cells and 3-4 background regions in each image, and the remaining 90% of images were automatically analyzed. ilastik probability maps were exported as cell masks. Finally, a CellProfiler pipeline was developed to quantify the number and size of macropinosomes per cell. The CellProfiler pipelines used for this study are available at GitHub (github.com/zmendel/Joel-Swanson-Lab). In brief, single cells were defined by propagation of nuclear objects based on Hoechst 33342 staining to the cell periphery as defined by ilastik cell masks. Macropinosomes were defined based on object segmentation of intracellular FDx signal. Macropinosome number and area were measured and related to individual cells. The pipeline was validated using the control conditions (±CSF1) before being blindly applied to the remainder of experimental conditions. The average frequency and size of macropinosomes per cell from at least four experiments are reported for each condition.

To quantify the levels of CSF1 receptor on single-cell basis using microscopy, we developed an automated image analysis pipeline using CellProfiler. In brief, single cells were defined by propagation of nuclear objects using DAPI to the cell periphery using Phalloidin-FITC. CSF1R was visualized using allophycocyanin anti-CSF1 receptor antibody (1:1000 dilution). The average intensity of CSF1R on a per cell basis was quantified. The pipeline was validated using the control condition (+CSF1).

### Statistical methods

Statistical analysis for all experiments was performed using GraphPad Prism software. At least three independent experiments were performed in all cases using cells from at least two different mice. In each graph, bars indicate mean±s.e.m. Analysis was carried out using two-tailed ratio paired *t*-tests for all experiments comparing the experimental condition with the PBS control. For experiments showing data relative to the PBS condition, statistics were applied using the raw values as opposed to the relative values, which are shown. *P*-values less than 0.05 were considered significant (*P<0.05, **P<0.01, ***P<0.001, ****P<0.0001).

## Supplementary Material

Supplementary information

Reviewer comments
