# Supplementary material for: Amino acids suppress macropinocytosis and promote release of CSF1 receptor in macrophages
Source: J Cell Sci. 2022 Feb 21;135(4):jcs259284. doi: 10.1242/jcs.259284 (PMC8919328; doi:10.1242/jcs.259284)
Supplement: Supplementary information [file joces-135-259284-s1.pdf]

Figure S1

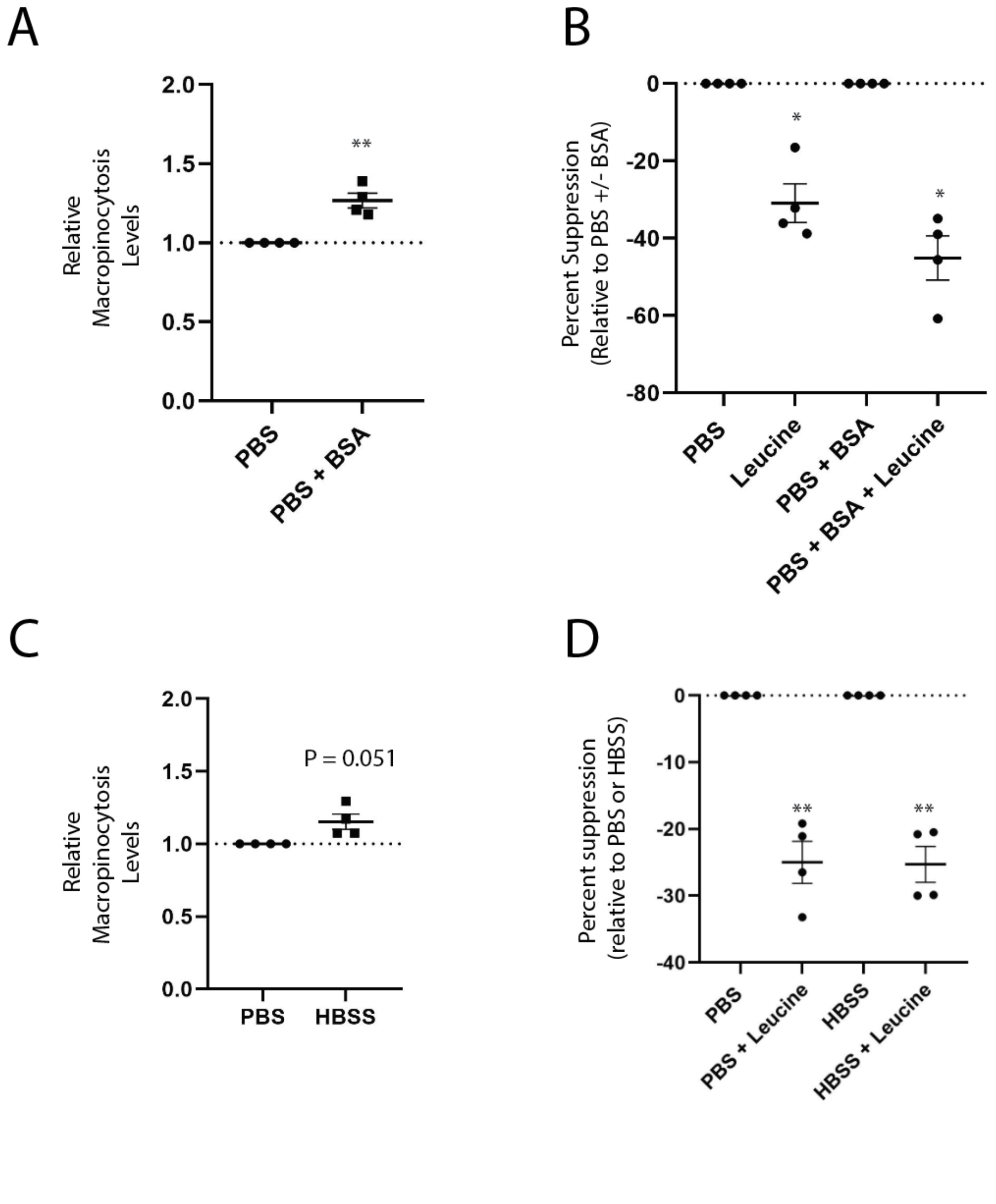

Journal of Cell Science • Supplementary information

Fig. S1. The effect of glucose and protein on leucine-induced suppression of macropinocytosis. (A-B) BMM were incubated for 30 min in PBS +/- 3% BSA in the presence or absence of leucine, then for 60 min with FDx and CSF1. Solute accumulation was analyzed by flow cytometry. **(A)** Relative macropinocytosis are determined by comparing the median fluorescence in the PBS + BSA condition to that in the PBS alone condition. **(B)** Percent suppression in the leucine conditions relative to those without leucine. Statistics are performed comparing conditions with leucine to their respective conditions without leucine **(C-D)** Cells were incubated for 30 min in either PBS or HBSS, with or without leucine, then 60 min with FDx and CSF1. Solute accumulation was analyzed by flow cytometry. **(C)** Relative levels of macropinocytosis in HBSS as compared to PBS. **(D)** Percent suppression in the leucine conditions relative to those without leucine. Statistics are performed comparing conditions with leucine to their respective conditions without leucine. N > 3 independent experiments. Each data point represents a single experiment. Bars indicate mean ± SEM. Statistics were performed using two-tailed ratio paired t tests for all experiments comparing the experimental condition to the PBS control, using the raw values as opposed to the relative values, which are shown. P values less than 0.05 were considered significant (*$p < 0.05$,**$p<0.01$)

Journal of Cell Science • Supplementary information

Figure S2

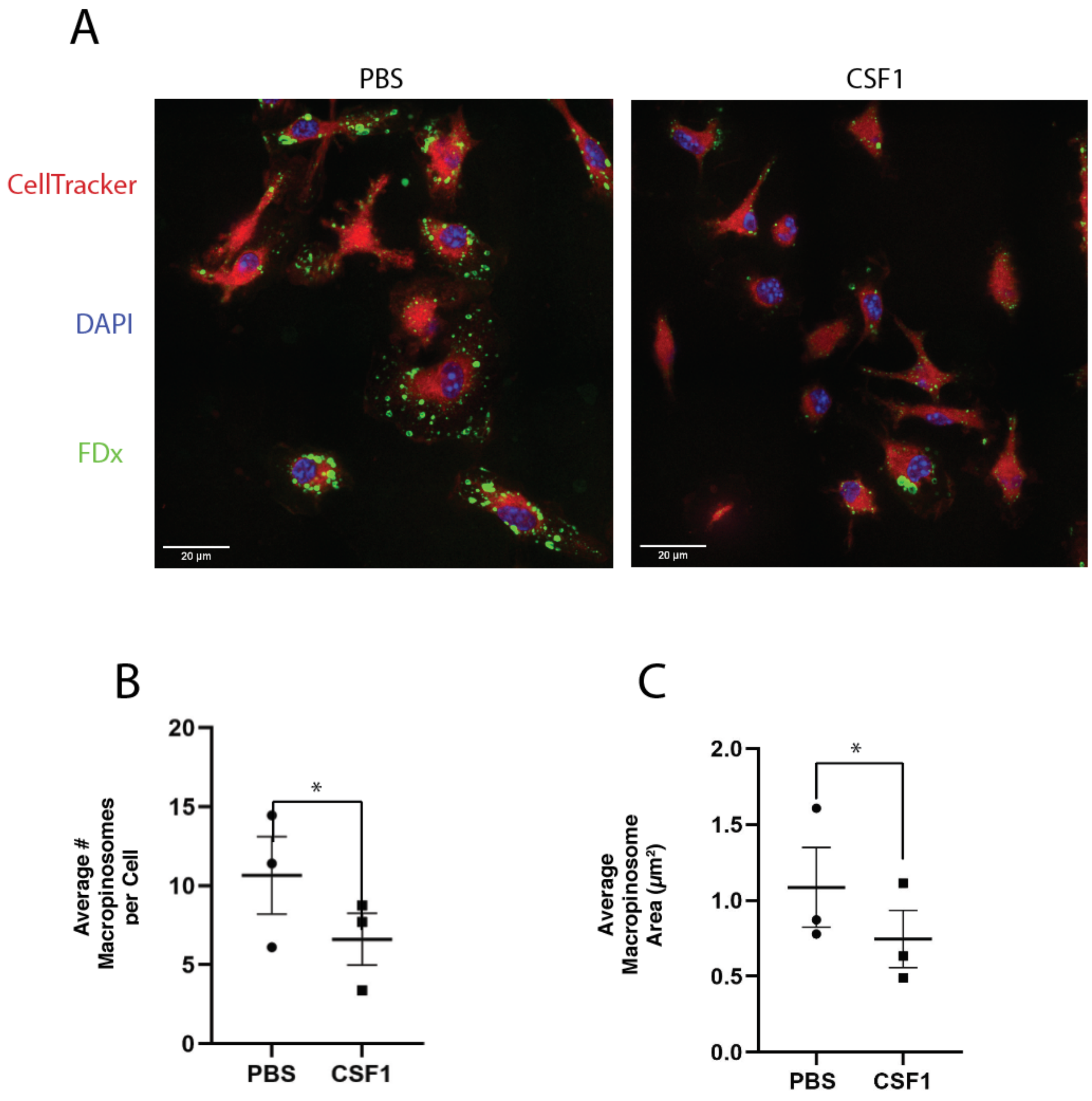
A
PBS
CSF1
CellTracker
DAPI
FDx
20 µm
20 µm
B
Average # Macropinosomes per Cell
20
15
10
5
0
*
PBS
CSF1
C
Average Macropinosome Area (µm²)
2.0
1.5
1.0
0.5
0.0
*
PBS
CSF1

Fig. S2. Downregulation of CSF1R by incubation in CSF1 results in fewer and smaller macropinosomes. (A-C) For the CSF1 condition, BMM were incubated in CSF1 in cell culture media lacking serum for 30 minutes, at which point both the PBS control cells and CSF1 cells were washed and incubated either in PBS alone for the control cells or PBS + CSF1 for the CSF1 condition. Following another 30-minute incubation, all cells were pulsed with FDx, CSF1, and Hoescht dye for 5 min. Cells were then fixed, stained with CellTracker™, and then mounted on coverslips. Cells were later imaged and analyzed using the CellProfiler™ pipeline. **(A)** Representative images showing CSF1R levels in macrophages incubated for 60 min in CSF1 or 30 min in buffer. **(B)** The average number of macropinosomes per cell is shown for the indicated conditions. **(C)** The average area of the macropinosomes in the indicated conditions. N = 3 independent experiments. Each data point represents a single experiment. Bars indicate mean ±SEM. Statistics were performed using two-tailed ratio paired t tests for all experiments comparing the experimental condition to the PBS control. P values less than 0.05 were considered significant (*$p < 0.05$).
