# Supplementary material for: Amino acids suppress macropinocytosis and promote release of CSF1 receptor in macrophages
Source: J Cell Sci. 2022 Feb 21;135(4):jcs259284. doi: 10.1242/jcs.259284 (PMC8919328; doi:10.1242/jcs.259284)
Supplement: Reviewer comments [file joces-135-259284_review_history.pdf]

Amino acids suppress macropinocytosis and promote release of CSF1 receptor in macrophages

Zachary I. Mendel, Mack B. Reynolds, Basel H. Abuaita, Mary X. O'Riordan and Joel A. Swanson
DOI: 10.1242/jcs.259284

Editor: Jennifer Lippincott-Schwartz

Review timeline

Original submission:	17 August 2021
Editorial decision:	27 September 2021
First revision received:	24 December 2021
Accepted:	31 December 2021

Original submission

First decision letter

MS ID#: JOCES/2021/259284

MS TITLE: Amino Acids Suppress Macropinocytosis and Promote Loss of CSF1 Receptor in Macrophages

AUTHORS: Zachary I Mendel, Joel A Swanson, Mary X O'Riordan, Mack B Reynolds, and Basel H Abuaita
ARTICLE TYPE: Research Article

We have now reached a decision on the above manuscript.

To see the reviewers' reports and a copy of this decision letter, please go to: https://submit-jcs.biologists.org and click on the 'Manuscripts with Decisions' queue in the Author Area. (Corresponding author only has access to reviews.)

As you will see, the reviewers raise a number of substantial criticisms that prevent me from accepting the paper at this stage. They suggest, however, that a revised version might prove acceptable, if you can address their concerns. If you think that you can deal satisfactorily with the criticisms on revision, I would be pleased to see a revised manuscript. We would then return it to the reviewers.

We are aware that you may be experiencing disruption to the normal running of your lab that makes experimental revisions challenging. If it would be helpful, we encourage you to contact us to discuss your revision in greater detail. Please send us a point-by-point response indicating where you are able to address concerns raised (either experimentally or by changes to the text) and where you will not be able to do so within the normal timeframe of a revision. We will then provide further guidance. Please also note that we are happy to extend revision timeframes as necessary.

Please ensure that you clearly highlight all changes made in the revised manuscript. Please avoid using 'Tracked changes' in Word files as these are lost in PDF conversion.

© 2022. Published by The Company of Biologists under the terms of the Creative Commons Attribution License (https://creativecommons.org/licenses/by/4.0/).

I should be grateful if you would also provide a point-by-point response detailing how you have dealt with the points raised by the reviewers in the 'Response to Reviewers' box. Please attend to all of the reviewers' comments. If you do not agree with any of their criticisms or suggestions please explain clearly why this is so.

Reviewer 1

Advance summary and potential significance to field

Macropinocytosis is increasingly thought of as a way for cells to obtain macromolecular food, which can be broken down and absorbed in the endo-lysosomal system. Macropinocytic feeding by cancer cells and also amoebae is well accepted, but its wider role in metazoan cells is still unfolding. A corollary is that food availability may regulate macropinocytosis.

With this in mind the present work is welcome. The authors ask whether macropinocytosis is regulated by amino acids in macrophages stimulated with CSF1. They found that any of a set of 9 mainly essential amino acids significantly repressed macropinocytosis, whereas the remaining, mainly non-essential amino acids were without effect. Oddly, the complete set of inhibitory and non-inhibitory amino acids was also without effect. Repression is likely be due to a reduction in CSF1 receptor numbers, brought about by an unknown but quite slow process. The experiments are clear-cut and have thoughtful controls, so the conclusions are both interesting and convincing.

Comments for the author

Having said that, the authors have restricted themselves to answering quite a narrow question. The distinguishing feature of macropinocytic feeding is that it allows cells to access proteins and other macromolecules, which are actually much more abundant by mass in serum than are amino acids. In contrast amino acids can be taken up both by macropinocytosis and by plasma membrane transporters. Therefore, it is important for the authors to show what effect proteins have on macropinocytosis in their system, either alone, or together with amino acids. For this experiment it might be important to test more than one protein to guard against retrieval from macropoinosomes before degradation.
Only a single amino acid concentration (0.25 mM) is checked, even though amino acids might vary greatly in potency and are present at very different concentrations in medium and serum. They should at least be checked at the concentrations used in RPMI-1640 medium.

Minor points
- what is the effect of glucose?
- any idea why the suppressive effect of amino acids should be specific to CSF1? Is it related to the different receptors and signal transduction pathways?
- the Dictyostelium case, where just three amino acids stimulate macropinocytosis is an interesting comparison: Williams, T.D., Kay, R.R., 2018. The physiological regulation of macropinocytosis during Dictyostelium growth and development. J. Cell Sci. 131, jcs.213736. And the strong regulation of macropinosome size mediated by the RasGAP, NF1 suggest that modulation of Ras activity is one potential way of controlling macropinosome size: Bloomfield, G. et al 2015. Neurofibromin controls macropinocytosis and phagocytosis in Dictyostelium. Elife 4, e04940. https://doi.org/10.7554/eLife.04940.
- some recent examples of what is likely to be macropinocytic feeding in coral, the gut, and across the placenta might be worth citing: Ganot, P., Tambutte, E. et al, 2020. Ubiquitous macropinocytosis in anthozoans. Elife 9, e50022; Hartenstein, V. & Martinez, P., 2019. Phagocytosis in cellular defense and nutrition: a food-centered approach to the evolution of macrophages. Cell Tissue Res. 377, 527–547; Shao et al, 2021. Placental trophoblast syncytialization potentiates macropinocytosis via mTOR signaling to adapt to reduced amino acid supply. Proc. Natl. Acad. Sci. U. S. A. 118, 2017092118.

© 2022. Published by The Company of Biologists under the terms of the Creative Commons Attribution License (https://creativecommons.org/licenses/by/4.0/).

Reviewer 2

Advance summary and potential significance to field

This study makes an interesting observation that certain amino acids, when added in the culture medium, are able to suppress macropinocytosis by primary mouse bone marrow macrophages stimulated with CSF1 or IL-34 (ligands of the CSF-1 receptor). These amino acids also decreased levels of CSF1-R and decreased the size of macropinosomes and amount of fluorescent dextran taken up by cells. The reported effects seem to be convincing, but the reason why amino acids suppress macropinocytosis or the mechanism(s) by which they do it are less clear. IT is also unclear how the levels of CSF1-R are reduced, other than that the receptor is degraded. While full mechanistic insight might be beyond the scope of this manuscript, some additional cell biology assays could shed light on mechanism. This might be achievable with some relatively straightforward imaging of the cells. Additionally, the decrease in size of macropinosomes is subtle and it isn't clear that this accounts for the decrease in uptake of dextran from the medium. In summary, this is an interesting study, but a little preliminary for JCS. Specific suggestions for revision are listed below.

Comments for the author

1. Can the authors provide live cell analysis of macropinocytosis taking place in the presence of the suppressive amino acids? How does frequency of events, ruffle size, cup size, actin dynamics compare with controls? This might help to explain why the macropinosomes are smaller as well as give insight into what effect the amino acids are having on macropinocytosis.
2. At what stage in the maturation process are the small macropinosomes shown in Figure 5 observed? This appears to be very early, as only 5 minutes of observations were carried out? Are these Rab5 positive structures at this stage?
3. Does inhibition of lysosomal degradation (e.g. chloroquine or primaquine) or proteasome inhibitors prevent loss of CSF1-R in the presence of leucine?
4. Can excess CSF1-R be detected in the culture supernatants of cells treated with the suppressive amino acids? This could support the idea that CSF1-R is somehow shed e.g. on extracellular vesicles.
5. Can CSF1-R mRNA be detected by Q-PCR and is this reduced upon addition of the suppressive amino acids?
6. Does reducing the level of CSF1-R by siRNA lead to reduced macropinosome size?

Reviewer 3

Advance summary and potential significance to field

Macropinocytosis is a mechanism for nutrient uptake by cells. Macrophages constitutively carry out macropinocytosis but it is significantly upregulated by the growth factor CSF-1, microbial products and other factors. However whether nutrients taken up by macropinocytosis regulate the process has not been studied in any detail. This manuscript uncovers a selective negative feedback mechanism whereby essential amino acids downregulate macropinocytosis in a CSF1R dependent manner, which is very interesting.

Comments for the author

As this is a short report, the mechanisms underpinning suppression of macropinocytosis and loss of surface CSF1R with exposure to essential amino acid are not fully elucidated. Nevertheless, the findings are important and deserve publicatrion.
However, several errors/concerns need correcting:
1. The statement in figure 3 legend that membrane permeabilization will allow visualization of intracellular immature CSF1R needs correcting as intracellular CSF1R is not cytoplasmic but membrane-bound and can in fact be seen in the perinuclear region in the first and fourth panels of Fig 3C.
2. While the essential amino acid-stimulated mechanism of receptor downregulation is clearly different to the rapid degradation induced by CSF-

© 2022. Published by The Company of Biologists under the terms of the Creative Commons Attribution License (https://creativecommons.org/licenses/by/4.0/).

1 the magenta CSF1R panels in figure 4B-C need ramping up so the endocytic distribution of CSF1R after CSF1 treatment and the slower more uniform receptor loss with amino acid treatment can be easily distinguished. They are currently not easily discernible, even at time 0.
3. A supplemental figure (S1) is referred to in the methods but is not available for review.
Typographical errors include:
Line 169 - "...rapid internalisation of (and) degradation of CSF1R.
Line 213 - "As the presence of TAMs are (is) associated with..."
Downregulation is sometimes written as down-regulation, e.g. line 231

First revision

Author response to reviewers' comments

Addressing Reviewer Comments:

Reviewer 1:

1) "what effect [do] proteins have on macropinocytosis in their system, either alone, or together with amino acids. For this experiment it might be important to test more than one protein, to guard against retrieval from macropinosomes before degradation.

- To address this, we performed flow cytometry based macropinocytosis experiments incubating the cells with or without 3% BSA to look at the effect of protein on macropinocytosis. We then incubated the cells +/- leucine in these conditions to see what effect BSA had on this suppressive effect by leucine. We observed that BSA significantly increased overall macropinocytosis but did not affect leucine-induced suppression of macropinocytosis. This data is introduced as a new figure, Figure S1, and is described on lines 88-91.

2) "Only a single amino acid concentration (0.25 mM) is checked, even though amino acids might vary greatly in potency and are present at very different concentrations in medium and serum. They should at least be checked at the concentrations used in RPMI-1640 medium. "

- The concentration of leucine in RPM1-1640 is 0.38mM. While we did not perform experiments at this concentration, we did perform a dose-response experiment looking at lower concentrations of leucine and what effect this has on macropinocytosis. We observed that maximal leucine-induced suppression is achieved at concentrations above 0.125mM. This data is shown in Figure 1D and is described on lines 110-115.

3) "what is the effect of glucose"

- We performed experiments looking at leucine-induced suppression using HBSS as the medium (5.55 mM glucose) instead of PBS (no glucose). We observed slightly increased overall macropinocytosis in conditions with glucose (albeit not statistically significant). We still observed leucine suppression when glucose was present. This data is included in Figure S1, and is described on lines 88-91.

4) "any idea why the suppressive effect of amino acids should be specific to CSF1? Is it related to the different receptors and signal transduction pathways?"

- We speculate that this is related to balancing the high energetic demands involved in cell growth. When the macrophage is in a nutrient-poor environment it will utilize energy undergoing macropinocytosis to bring in nutrients for survival. However, when it receives a signal to grow from CSF1, it now must expend energy for growth. It balances these energetic demands by prioritizing essential amino acids, dampening down macropinocytosis once sufficient levels of essential amino acids have been met. This may

© 2022. Published by The Company of Biologists under the terms of the Creative Commons Attribution License (https://creativecommons.org/licenses/by/4.0/).

conserve energy for growth-related activities. We are designing experiments to test this hypothesis.

5) "The Dictyostelium case, where just three amino acids stimulate macropinocytosis is an interesting comparison: Williams, T.D., Kay, R.R., 2018. The physiological regulation of macropinocytosis during Dictyostelium growth and development. J. Cell Sci. 131, jcs.213736. And the strong regulation of macropinosome size mediated by the RasGAP, NF1 suggest that modulation of Ras activity is one potential way of controlling macropinosome size: Bloomfield, G. et al 2015. Neurofibromin controls macropinocytosis and phagocytosis in Dictyostelium. Elife 4, e04940. https://doi.org/10.7554/eLife.04940."

- Thank you for these suggestions. We now cite the Kay 2018 paper in the introduction and the Bloomfield 2015 paper in the discussion. These can be found on lines 68-69 and lines 287-288 respectively.

6) "Some recent examples of what is likely to be macropinocytic feeding in coral, the gut, and across the placenta might be worth citing: Ganot, P., Tambutte, E. et al, 2020. Ubiquitous macropinocytosis in anthozoans. Elife 9, e50022; Hartenstein, V. & Martinez, P., 2019. Phagocytosis in cellular defense and nutrition: a food-centered approach to the evolution of macrophages. Cell Tissue Res. 377, 527–547; Shao et al, 2021. Placental trophoblast syncytialization potentiates macropinocytosis via mTOR signaling to adapt to reduced amino acid supply. Proc. Natl. Acad. Sci. U. S. A. 118, 2017092118."

- Thank you for bringing these papers to our attention. We found the last paper quite relevant and included a line in the discussion about this. This is found on lines 274- 275.

Reviewer 2:

1) "Can the authors provide live cell analysis of macropinocytosis taking place in the presence of the suppressive amino acids? How does frequency of events, ruffle size, cup size, actin dynamics compare with controls? This might help to explain why the macropinosomes are smaller as well as give insight into what effect the amino acids are having on macropinocytosis."

- This is an interesting question but one which seems unlikely to yield significant relevant insights to this phenomenon without extensive morphometry. We have added the following text to the Results (lines 209-212): "In live cell imaging of cells incubated in CSF1 vs CSF1 + leucine, we could not discern any obvious differences in ruffling or the process of macropinosome formation. This was likely due to the wide range of morphologies that characterize macropinocytosis (Quinn, et al., Nature Commun. 2021)."

2) "At what stage in the maturation process are the small macropinosomes shown in Figure 5 observed? This appears to be very early, as only 5 minutes of observations were carried out? Are these Rab5 positive structures at this stage?"

- We have added the following text to the Results section describing Figure 6 (lines 215-219): "Because macropinosomes shrink and fuse shortly after closing into the cell, it was necessary to image them for morphometry after only brief pulses with FDx to best approximate their initial sizes. Earlier work from this lab (Racoosin, JCB 1993) and others (Maxson, JCS 2020) showed that 1-5-minute pulsed macropinosomes are enriched in markers of early endosomes including Rab5."

3) "Does inhibition of lysosomal degradation (e.g. chloroquine or primaquine) or proteasome inhibitors prevent loss of CSF1-R in the presence of leucine?"

- We have added new data and a new figure (Figure 4) showing that inhibition of lysosomal degradation by Bafilomycin A1 did not prevent loss of CSF1R in the presence of leucine. As

© 2022. Published by The Company of Biologists under the terms of the Creative Commons Attribution License (https://creativecommons.org/licenses/by/4.0/).

a positive control we incubated cells in PBS + CSF1. CSF1 is known to promote lysosomal degradation of CSF1R. Baf prevented CSF1R loss in the CSF1 condition. These experiments are described on lines 175-185.

4) “Can excess CSF1-R be detected in the culture supernatants of cells treated with the suppressive amino acids? This could support the idea that CSF1-R is somehow shed e.g. on extracellular vesicles.”

- In additional new experiments, we show that CSF1R is enriched in the medium of cells incubated in leucine. Moreover, the amount we detected was significantly higher than what appeared in the medium of cells incubated in PBS alone or with the non- suppressive amino acid serine. This data is included in the new Figure 4, and is described on lines 186-192.

5) “Can CSF1-R mRNA be detected by Q-PCR and is this reduced upon addition of the suppressive amino acids?”

- This interesting mechanistic question is beyond the scope of the present study, which demonstrates inhibition of CSF1-stimulated macropinocytosis through specific loss of CSF1R. This and additional mechanistic questions are being addressed in follow-up studies.

6) “Does reducing the level of CSF1-R by siRNA lead to reduced macropinosome size?”

- Instead of using siRNA, we lowered CSF1R levels by pre-incubating cells in CSF1, which has been shown here and elsewhere to reduce CSF1R levels by degradation in lysosomes. After preincubation in CSF1, macrophages made fewer and smaller macropinosomes in response to additional CSF1. This data is included in Figure S2 and is described on lines 225-231.

Reviewer 3:

1) “The statement in figure 3 legend that membrane permeabilization will allow visualization of intracellular immature CSF1R needs correcting as intracellular CSF1R is not cytoplasmic but membrane-bound and can in fact be seen in the perinuclear region in the first and fourth panels of Fig 3C. “

- Thank you for this comment. We changed the text to reflect this by stating (lines 661-662), “cells were permeabilized and stained using anti-CSF1R receptor antibody to visualize total CSF1R”

2) “While the essential amino acid-stimulated mechanism of receptor downregulation is clearly different to the rapid degradation induced by CSF-1, the magenta CSF1R panels in figure 4B-C need ramping up so the endocytic distribution of CSF1R after CSF1 treatment and the slower more uniform receptor loss with amino acid treatment can be easily distinguished. They are currently not easily discernible, even at time 0.”

- This figure is now Figure 5. Increasing the fluorescence signal in panel 5B to visualize CSF1R dynamics in response to CSF1 addition would have allowed better visualization of CSF1R dynamics, but applying that same intensity increase to the comparable PBS and leucine conditions would have saturated the PBS and leucine images, making them unintelligible. Instead, we increased the signals in 5C and greatly increased the CSF1R signal in the CSF1 condition. This allowed better visualization of the endocytic dynamics of CSF1R in this condition compared to the dynamics of CSF1R in the leucine or PBS conditions.

© 2022. Published by The Company of Biologists under the terms of the Creative Commons Attribution License (https://creativecommons.org/licenses/by/4.0/).

A supplemental figure (S1) is referred to in the methods but is not available for review.

- We thank the reviewer for spotting this oversight. While there previously were not supplemental figures in the original submission, there are new supplemental figures in this revised submission.

3) Typographical errors include:

Line 169 - "…rapid internalisation of (and) degradation of CSF1R.
Line 213 - "As the presence of TAMs are (is) associated with…"
Downregulation is sometimes written as down-regulation, e.g. line 231

- We thank the reviewer for catching these typographical errors and have made these corrections.

Second decision letter

MS ID#: JOCES/2021/259284

MS TITLE: Amino Acids Suppress Macropinocytosis and Promote Release of CSF1 Receptor in Macrophages

AUTHORS: Zachary I Mendel, Mack B Reynolds, Basel H Abuaita, Mary X O'Riordan, and Joel A Swanson
ARTICLE TYPE: Research Article

I am happy to tell you that your manuscript has been accepted for publication in Journal of Cell Science, pending standard ethics checks.

© 2022. Published by The Company of Biologists under the terms of the Creative Commons Attribution License (https://creativecommons.org/licenses/by/4.0/).
